# Three-Dimensional Array Interpolation Imaging Algorithm of Water Holdup by the Capacitance Array Tool of Oil–Water Two-Phase Flow in Horizontal Wells

**DOI:** 10.3390/s26041388

**Published:** 2026-02-23

**Authors:** Doujuan Zhang, Haimin Guo, Yongtuo Sun, Aibing Fu, Ao Li, Dudu Wang, Yuqing Guo, Mingyu Ouyang, Liangliang Yu, Wenfeng Peng

**Affiliations:** 1 College of Geophysics and Petroleum Resources, Yangtze University, Wuhan 430100, China or zhangdoujuan.slyt@sinopec.com (D.Z.); 2022720524@yangtzeu.edu.cn (Y.S.); 2022730026@yangtzeu.edu.cn (A.L.); 2023720526@yangtzeu.edu.cn (D.W.); 2023710415@yangtzeu.edu.cn (Y.G.); 2024720502@yangtzeu.edu.cn (M.O.); 2024710438@yangtzeu.edu.cn (L.Y.); 2024710433@yangtzeu.edu.cn (W.P.); 2 Research Institute of Exploration and Development, Shengli Oilfield Company, Sinopec, Dongying 257015, China; fuaibing.slyt@sinopec.com; 3 Key Laboratory of Exploration Technologies for Oil and Gas Resources, Yangtze University, Ministry of Education, Wuhan 430100, China

**Keywords:** horizontal wells, Capacitance Array Tool, oil–water two-phase flow, three-dimensional visualization, interpolation algorithms

## Abstract

Due to the gravitational differentiation effect, the oil–water two-phase flow in the horizontal well exhibits significant asymmetry and inhomogeneity in terms of phase distribution and velocity field. The existing logging techniques are difficult to use to precisely characterize the wellbore flow field under these conditions. To solve this problem, this study, based on the logging data of the Capacitance Array Tool, proposes a three-dimensional visualization method for the water holdup field in the wellbore and applies and evaluates three interpolation algorithms: linear interpolation, cubic spline interpolation, and natural neighbor interpolation. This paper relies on the multiphase flow experimental platform and uses industrial white oil and tap water as fluid media for experiments. It systematically studies the three-dimensional imaging characteristics under different angles, flow rates, and water cuts. The results show that the natural neighbor interpolation algorithm, with its advantage in topological reconstruction, effectively overcomes local mutations in complex flow states. It exhibits superior imaging accuracy and robustness under all operating conditions but has higher computational costs. In contrast, linear interpolation and cubic spline interpolation perform well only in stable flow fields with low-to-moderate flow rates and water holdup. In practical applications, for simple flow states, it is recommended to use computationally efficient linear or cubic spline interpolation methods; for complex flow states or scenarios requiring strict imaging details, the natural neighbor interpolation algorithm should be prioritized.

## 1. Introduction

The large-scale development of horizontal wells and near-horizontal wells has created an urgent need for production logging technologies. Compared to vertical wells, the geometric complexity of non-vertical wells has significantly increased [1]. Conventional logging instruments are difficult to use effectively in the confined space of the well. Especially in the later stages of development, the continuous increase in water cut leads to a dramatic increase in the difficulty of predicting the multiphase flow state in the well, urgently requiring breakthroughs in the production logging technology for horizontal wells. The accurate measurement of water holdup is a core component of production profile interpretation. By using high-precision water holdup data and combining advanced imaging technology, the three-dimensional analysis of the distribution law of wellbore phases and overall water holdup characteristics can be achieved, providing a key theoretical basis for optimizing production plans and formulating efficient oil extraction processes.

Accurate water holdup measurement is the prerequisite for wellbore fluid imaging. A review of the literature over the past few decades reveals that most methods utilize the significant difference in conductivity between oil and water. Various conductance sensors [2,3,4,5,6,7,8,9,10,11] measuring the current conduction capability of mixed fluids are widely used. Wang et al. [12] proposed a new method for measuring the water holdup in horizontal wells with two-phase oil–water flow by combining a circular array conductance sensor with a circumferential array conductance probe sensor. The method was verified through simulation experiments. Under the conditions of a total flow rate ranging from 10 to 200 m^3^/d, water cut from 30% to 90%, and five different inclinations, the absolute value of the measurement error was less than 5%. Additionally, microwave sensors [13,14,15,16], as well as combination tools integrating microwave, capacitance, and conductance sensors [17,18], have been employed. Furthermore, artificial intelligence algorithms are increasingly applied to predict liquid holdup in mixed fluids [19,20,21]. Evidently, accurate measurement is a critical step in production logging interpretation.

Imaging results of water holdup directly characterize the phase distribution of multiphase fluids in the wellbore cross-section. Wang et al. [22] proposed a multiphase flow visualization method based on ERT-ECT dual-modal electrical tomography, which realizes the characterization of the phase distribution of the six common flow types of gas, oil, and water in the horizontal pipeline, but it only focuses on the visualization of the phase distribution in two-dimensional cross-sections. Addressing the significant measurement errors of traditional instruments under stratified flow conditions, this study utilizes the Capacitance Array Tool (CAT), jointly developed by Halliburton and Sondex. The application of this tool has driven progress in water holdup imaging technology. Dai et al. [23] conducted experiments using CAT at a deviation of −30°, a total flow rate of 100 m^3^/d, and water cuts ranging from 20% to 80%, employing Gaussian Radial Basis Functions (GRBF) to image the cross-sectional water holdup. Their results showed that the algorithm could comprehensively resolve CAT response data, revealing the coupling effects of flow rate, water cut, and deviation angle, though instrument rotation was not considered. Dong et al. [24,25] applied improved distance factor imaging algorithms and rotation-corrected GRBF to three-phase flow data (90° deviation), verifying that these methods could accurately reconstruct fluid stratification even with instrument rotation. Wu et al. [26] compared linear, cubic spline, inverse distance weighting, and GRBF algorithms across low, medium, and high flow rates, establishing criteria for stratified flow: linear or cubic spline is preferred for low flow-low water cut conditions, while GRBF is necessary for low flow-high water cut conditions.

While previous studies have extended to medium-high flow rates, imaging dimensions have largely been limited to 2D cross-sections. Although 2D imaging [27,28] improves interpretation accuracy, it fails to characterize the dynamic evolution of fluids along the wellbore axis, hindering the precise analysis of complex flow structures (e.g., vertical migration of phase distribution, axial flow regime transition). 3D water holdup imaging has become a frontier direction to break through existing interpretation bottlenecks, requiring the integration of high-precision array sensors with spatial interpolation algorithms to achieve full-dimensional visualization of wellbore multiphase flow.

In this paper, we first conducted the fluid flow experiments with an inclination angle of 90°, flow rates of 100 m^3^/d, 300 m^3^/d, and 600 m^3^/d, and water cuts of 20%, 40%, 60%, and 90%. After the end of the experiment, we intercepted the wellbore data after the turbine flow curve was stabilized. Starting from the initial section, we obtained the water holdup data of the section every 0.1 m, and a total of 1440 data points were obtained. Then, we use linear interpolation, cubic spline interpolation, and natural neighbor interpolation to carry out three-dimensional interpolation imaging of the water holdup of the obtained data points and conduct a comparative analysis.

The main contributions of this paper are as follows:

This paper conducts experiments on horizontal well oil–water two-phase flow under different flow rates and water cut conditions, systematically compares the water holdup imaging accuracy, stability, and adaptability of linear interpolation, cubic spline interpolation, and natural neighbor interpolation imaging algorithms, and clarifies the optimal matching criteria of the algorithms under different working conditions. At the same time, it is found that in high-flow conditions, the existing single-data three-dimensional imaging is prone to distortion and difficult to characterize complex mixed-phase states. Therefore, it is proposed that subsequent research adopt the technical path of multi-source data coupling or introducing artificial intelligence algorithms to achieve refined three-dimensional imaging of wellbore fluids under high-flow conditions, providing a new direction for complex flow field visualization.

The structure of this paper is as follows: Section 1 is the introduction, which elaborates on the importance of precise water holdup measurement in horizontal wells for reservoir development dynamic monitoring and reviews the research progress, current situation, and limitations of two-dimensional imaging technology of wellbore cross-sections. Section 2 introduces the horizontal well multiphase flow experimental setup, the working principle, and the parameter configuration of the array capacitance water holdup meter, as well as the characteristic analysis of typical flow patterns in the experiments. Section 3 focuses on the processing of measurement data, including the analysis of raw data and the data screening strategy in the stable flow field stage, providing a reliable data basis for subsequent imaging analysis. Section 4 systematically expounds the basic principles and mathematical models of the three interpolation algorithms. Section 5 analyzes the imaging results in combination with the flow mechanism, obtains the algorithm matching criteria under different working conditions, and based on the imaging limitations in high-flow conditions, proposes the subsequent research direction of multi-source data coupling and integration of artificial intelligence algorithms.

## 2. Experimental Work

### 2.1. Experimental Equipment

**Figure 1:**
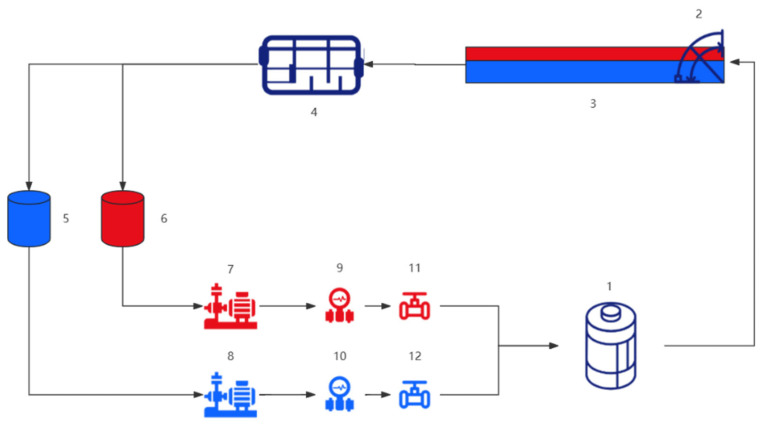
Multiphase flow experimental system simulation diagram. (1) Oil–water mixer; (2) Inclination regulator; (3) Transparent simulated wellbore (12 m length, 159 mm inner diameter); (4) Oil–water separator; (5/6) Water/Oil tanks; (7/8) Oil/Water pumps; (9/10) Flowmeters; (11/12) Control valves.

Experiments were designed and conducted at the Multiphase Flow Laboratory of Yangtze University. The facility has hosted numerous multiphase flow research projects, including simulations for horizontal well instruments such as CAT, RAT, SAT, and conventional production logging tools. The experimental media were industrial white oil, air, and tap water. The simulated liquid flow range is 0–600 m^3^/d, and the gas flow range is 0–1000 m^3^/d. The setup is illustrated in Figure 1.

**Figure 2:**
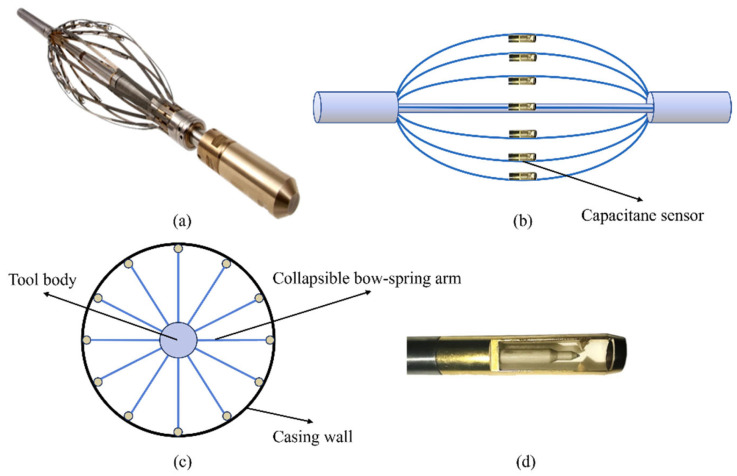
Physical diagram of the CAT instrument, sensor, and cross-section schematic. (**a**) The physical diagram of the instrument (**b**) the side view diagram of the instrument (**c**) The specific arrangement of the sensors in the wellbore (**d**) the capacitance sensor probe.

The Capacitance Array Holdup Meter (CAT), developed in 1999 by the UK-based Sondex Company (now a part of Baker Hughes), arranges 12 holdup probes around the wellbore through the collapsible bow-spring arms. The physical diagram of the instrument is shown in Figure 2a, the side view diagram of the instrument is in Figure 2b, and the capacitance sensor probe is shown in Figure 2d. The specific arrangement of the sensors in the wellbore is depicted in Figure 2c.

**Figure 3:**
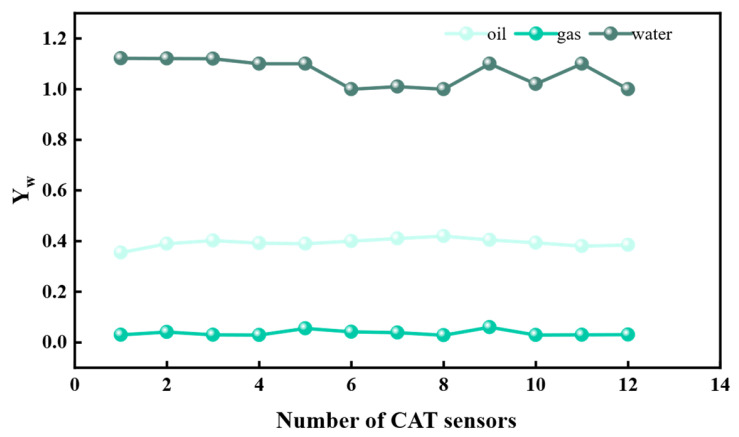
Response values of CAT sensors in oil, gas, and water phases.

This instrument achieves phase state identification based on the differences in dielectric constants of oil, gas, and water. The dielectric constant of gas is generally 1, that of water is generally 80, and the dielectric constant of oil depends on the oil itself. The 12 sensors of the instrument are installed on the support arm and simultaneously measure the fluid capacitance in the near-wellbore area of a single section. It can collect over 100 frames of data per second, and the data can be transmitted in real time to the ground or stored in the instrument’s memory, providing raw data for subsequent three-dimensional interpolation imaging. Before the experiment, the sensors need to be calibrated for responses to the three phases of oil, gas, and water. Figure 3 shows the response values of the sensors in a single-phase fluid. The results show that the 12 probes all respond with high values to the water phase, the response value of the oil phase is approximately 0.4, and the response value of the gas phase is close to 0.

### 2.2. Experimental Scheme

Following calibration, multiphase flow experiments were conducted under varying deviations, water cuts (Cw), and measurement modes (stationary vs. logging). The logging scheme for oil–water two-phase flow is detailed in Table 1. An electric winch positioned the tool within the transparent wellbore. The console controlled total flow and water cut, while the inclination regulator adjusted the wellbore angle.

This paper primarily focuses on 3D interpolation imaging of oil–water two-phase flow in a horizontal well (90°) with a logging speed of 10 m/min.

### 2.3. Oil–Water Two-Phase Flow Regime Analysis

**Figure 4:**
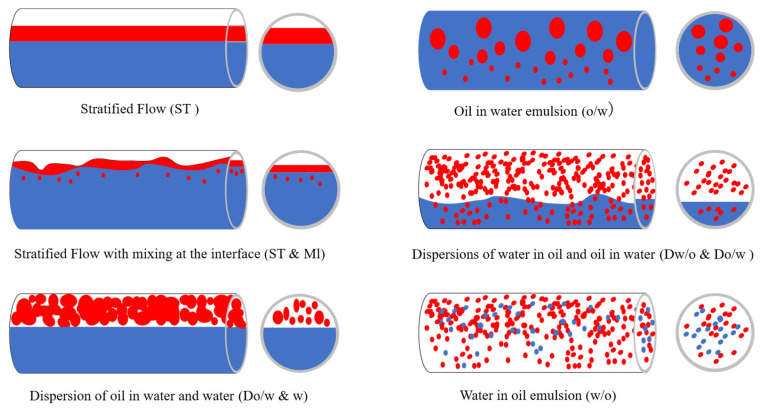
Oil–water two-phase flow regimes in horizontal wellbores. (The blue color represents water, and the red color represents oil).

To validate the 3D interpolation results, it is essential to analyze the flow regime evolution. Influenced by flow rate and water cut, the flow in the wellbore exhibits significant polymorphism. Based on prior research [29], six flow regimes are identified in horizontal oil–water flows, as shown in Figure 4: stratified flow (ST); stratified flow with mixing at the interface (ST & MI); dispersion of oil in water and water (Do/w & w); oil in water emulsion (o/w); dispersions of water in oil and oil in water (Dw/o & Do/w); water in oil emulsion (w/o).

In this experiment, in order to correspond with the experimental results and obtain a more intuitive description of the flow patterns, the image data retained during the experiment were comprehensively analyzed based on the theoretical model shown in Figure 4, and the formation mechanisms of various fluid flow patterns during the experiment were studied. The final analysis results are as follows:

Low Flow: Density differences and immiscibility lead to gravitational segregation, forming smooth stratified flow (ST). As flow increases, velocity differences generate small oil bubbles, creating stratified flow with mixing interface (ST & MI). Further aggregation of bubbles forms large buoyant structures, resulting in horizontal bubble flow (Do/w & w).

High Flow: High velocity disperses oil bubbles throughout the wellbore (o/w). When significant gas bubbles or droplets exist at the interface under agitation, stable thin films form, creating foam/froth flow (Dw/o & Do/w). Under further mixing and gravity effects, droplets merge into a uniform emulsion (w/o) where no distinct interface exists.

## 3. Measurement Data Analysis

**Figure 5:**
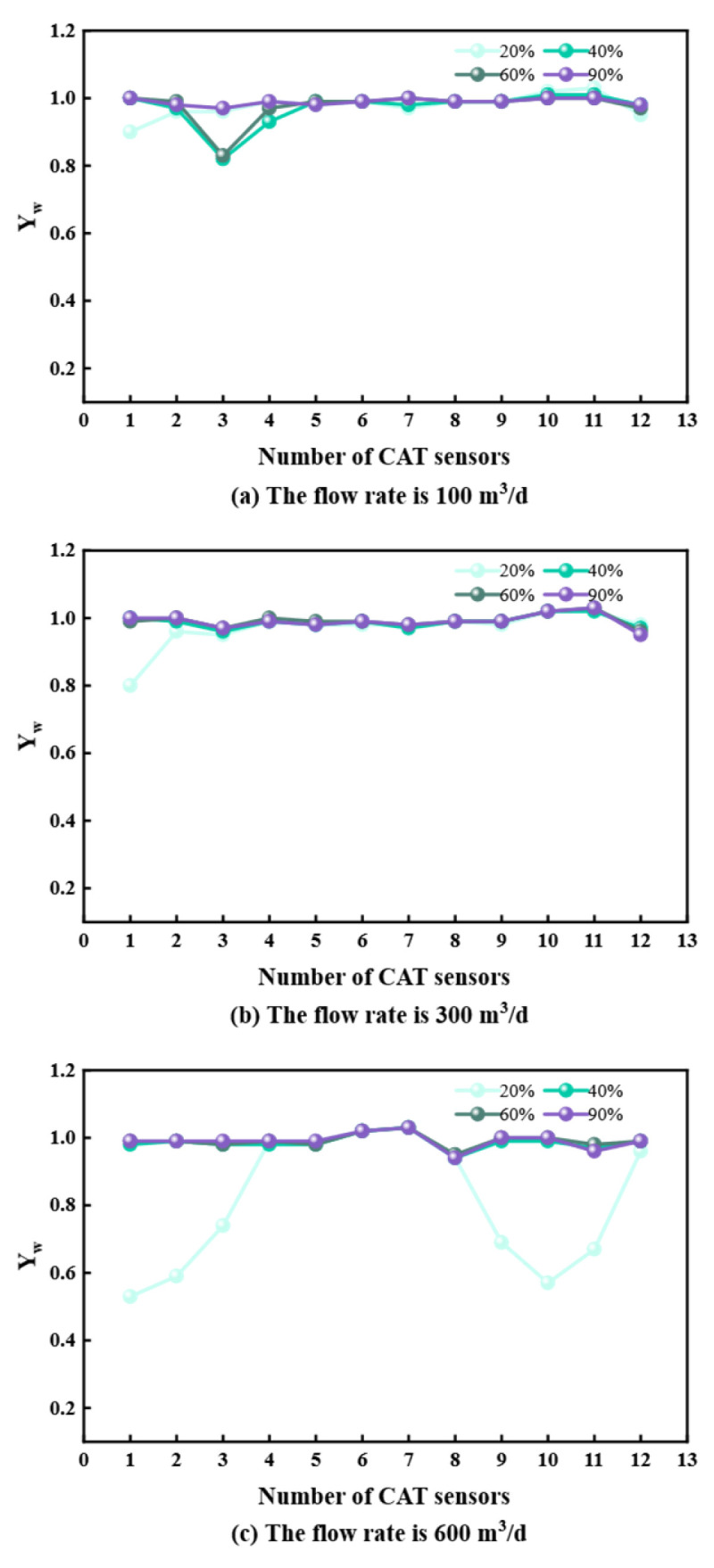
CAT response at different flow rates at 60° inclination.

**Figure 6:**
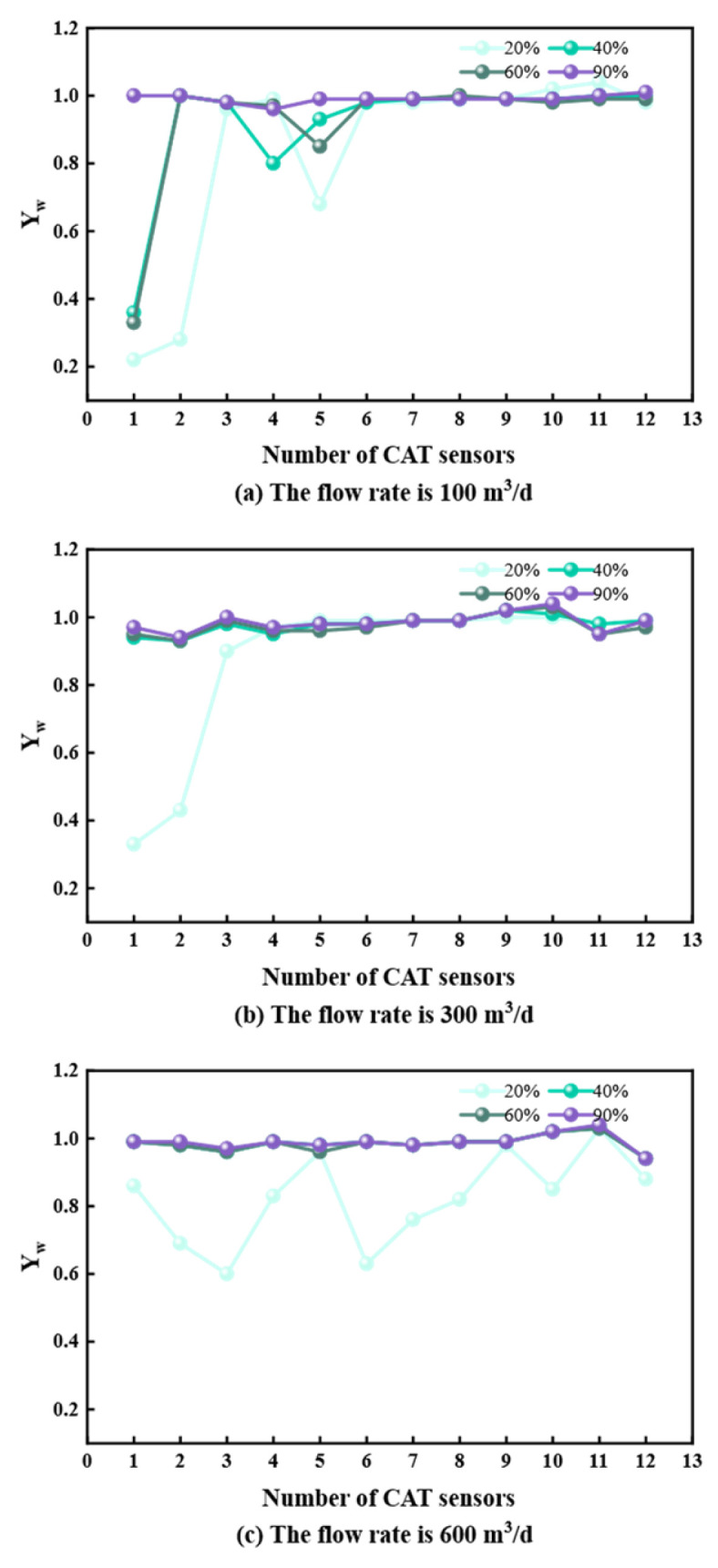
CAT response at different flow rates at 85° inclination.

**Figure 7:**
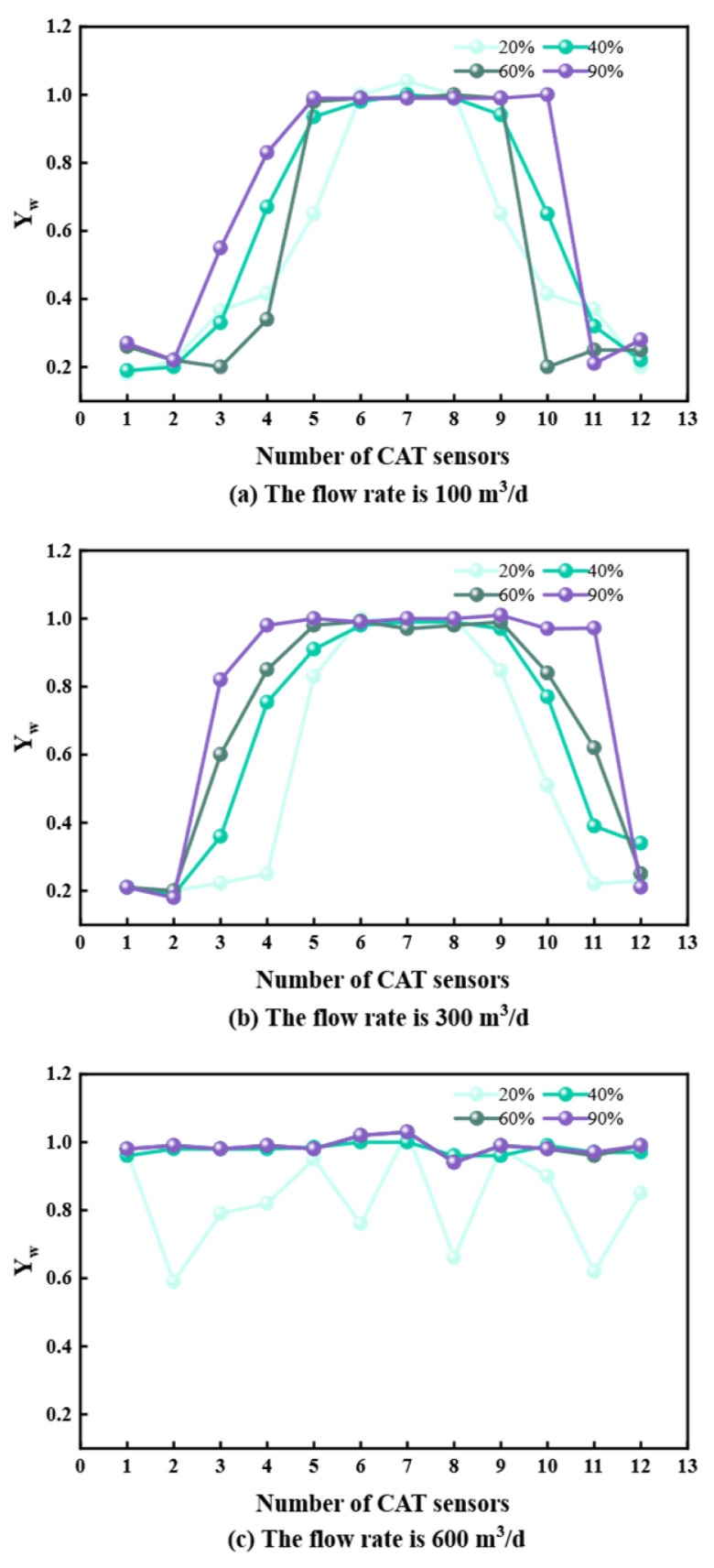
CAT response at different flow rates at 90° inclination.

**Figure 8:**
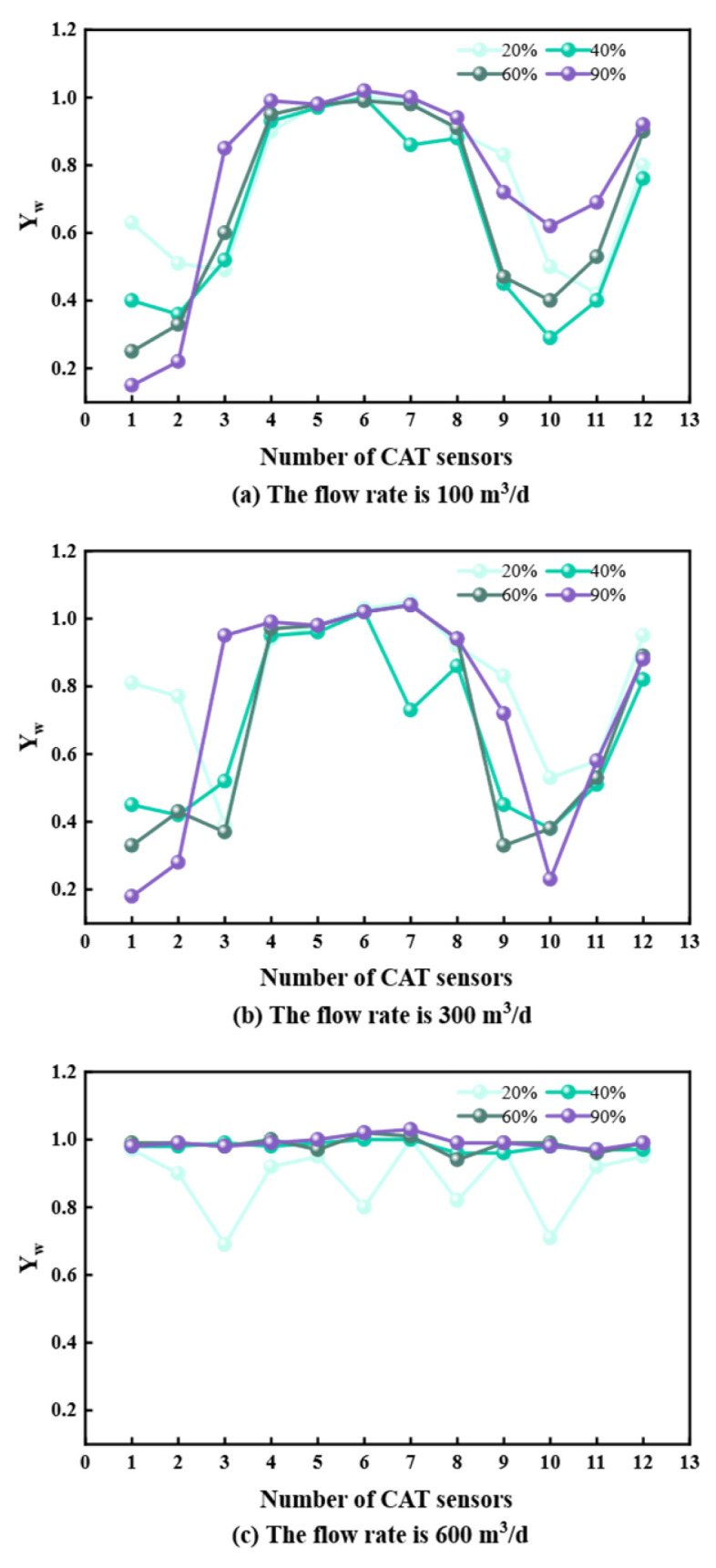
CAT response at different flow rates at 93° inclination.

To characterize flow properties, stable flow sections were analyzed for deviations of 60°, 85°, 90°, and 93° at flow rates of 100, 300, and 600 m^3^/d. Data from the 12 CAT sensors were extracted to analyze the flow–water cut–phase distribution relationship (Figure 5, Figure 6, Figure 7 and Figure 8).

At 60° and 85° deviations (Figure 5 and Figure 6), oil and water undergo upward flow. At 20% water cut, lower sensors detect oil; as water cut increases, water displaces oil to the center, leading to higher water readings. At 90° and 93° deviations (Figure 7 and Figure 8) with 100 and 300 m^3^/d flow rates, gravity segregation dominates, forming ST and ST & MI regimes. Water flows stably at the bottom, oil at the top. Sensor responses show a strong correlation with vertical spatial distribution, verifying CAT’s accuracy in stratified flows. However, at 600 m^3^/d, the flow transitions to foam and emulsion regimes. Phase dispersion causes systematic deviations in sensor response. Aside from fluctuations at 20% water cut due to discrete oil phase, sensors at 40%, 60%, and 90% water cuts are almost entirely covered by the continuous water phase, deviating significantly from the actual phase distribution.

The stabilization distance of different flow regimes varies with their hydrodynamic characteristics. For stratified flow, characterized by a stable flow field, the turbine flow curve stabilizes at approximately 4 m from the inlet. In contrast, for flow regimes with intense turbulence (e.g., foam flow), the flow curve does not stabilize until around 9 m from the inlet. To mitigate interference from transient data during flow regime adjustment on interpolation results, only wellbore data collected after the turbine flow curve stabilizes were used in this study. This ensures that the input data for the interpolation algorithms accurately reflects the phase distribution characteristics of the stable flow field.

Considering the axial variation features of different flow phenomena and the dynamic response characteristics of the sensors, an axial sampling interval of 0.1 m was determined. For stable flow regimes, the axial phase distribution changes gently, and a 0.1-meter interval is sufficient to capture spatial variations comprehensively. For high-turbulence flow regimes, despite the intense fluctuations in axial phase distribution, a 0.1-meter interval still ensures no critical topological changes (e.g., abrupt interface transitions) are overlooked. Furthermore, computational cost increases exponentially as the sampling interval decreases. Thus, the 0.1-meter sampling interval adopted in this study represents an optimal balance between imaging accuracy and computational efficiency.

## 4. Interpolation Imaging Algorithms

The geometric foundations of the interpolation algorithms discussed are Delaunay Triangulation and Voronoi Polygons [30]. Accuracy depends heavily on the definition of spatial topology.

### 4.1. Voronoi Diagram & Delaunay Triangulation

The Voronoi diagram defines the “region of influence” for sampling points. For a set of discrete points $P=\left\{p_{1},p_{2},\ldots ,p_{n}\right\}$, the Voronoi cell $V_{i}$ of any point $p_{i}$ is defined as the set of all points on the plane that are closer to $p_{i}$ than to any other point $p_{j}$:

$$V_{i}=\{x\in R^{2}|\|x-p_{i}\|<\|x-p_{j}\|,\forall j\neq i\}$$

In a physical sense, the Voronoi diagram divides the continuous space into multiple polygonal regions, and the attribute values within each region are most influenced by the central sampling point of that region. It serves as the geometric foundation for nearest neighbor search and natural neighbor interpolation.

**Figure 9:**
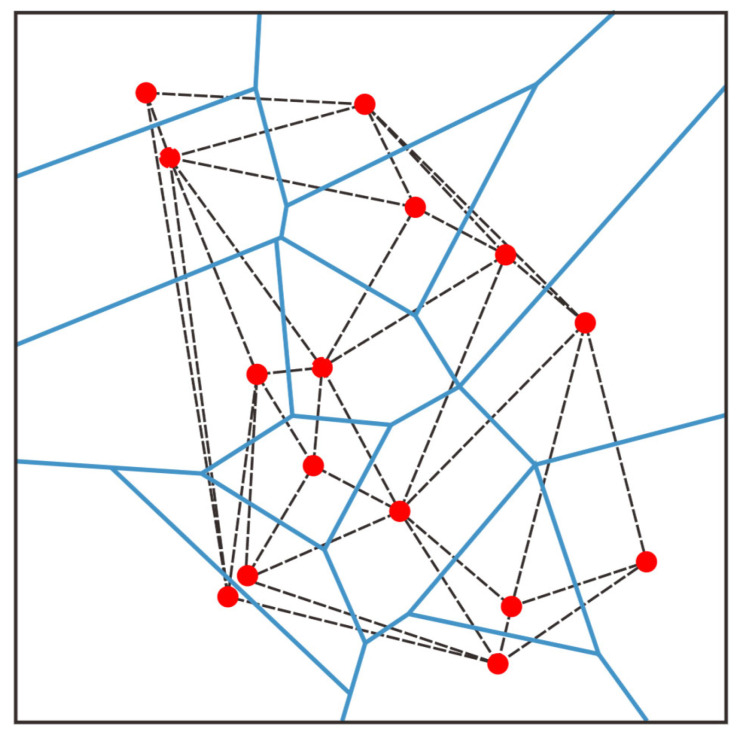
Duality of Delaunay & Voronoi (broken line represents Delaunay; solid blue line represents Voronoi, red bullet represents discrete point).

Delaunay triangulation is the dual graph of the Voronoi diagram [31], as shown in Figure 9. It is a triangular mesh constructed by connecting the generating points of all adjacent Voronoi cells. Delaunay triangulation has a crucial “empty circle property”: the circumscribed circle of any triangle in the triangulation does not contain any other data points from the point set P. This property ensures that the generated triangles are as close to “equilateral” shapes as possible, avoiding the appearance of narrow triangles, thereby maximizing the numerical stability of the interpolation calculation. It is the geometric basis for linear interpolation and cubic spline interpolation.

### 4.2. Linear Interpolation

Based on Delaunay triangulation, linear interpolation fits a plane (Equation (1)) to the three vertices of each triangle:

(1) $$f\left(x,y\right)=Ax+By+C$$

This method guarantees $C^{0}$ continuity. It is computationally efficient and strictly convex (results stay within the min/max range of data) but produces faceted, angular surfaces.

### 4.3. Cubic Spline Interpolation

Also based on Delaunay triangulation, this method fits a 2D cubic polynomial surface to each triangle, ensuring continuity of first-order partial derivatives ($C^{1}$ or $C^{2}$). While it produces smooth surfaces, it may generate non-physical oscillations (overshoot) in areas with steep gradients, such as gas–liquid interfaces [32].

### 4.4. Natural Neighbor Interpolation

**Figure 10:**
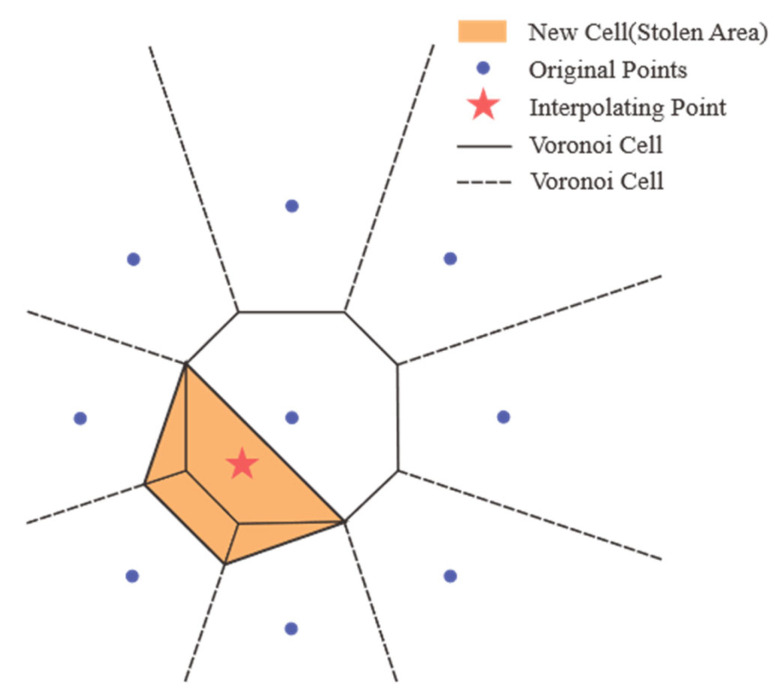
Natural neighbor principle.

Natural neighbor (Sibson) interpolation is based on Voronoi geometry. For an interpolation point q, the algorithm simulates inserting q into the point set and generating a new Voronoi cell. The value is a weighted average where weights $\omega_{i}$ are proportional to the overlap area borrowed from neighboring cells (Figure 10).

In the figure, the red star represents the point q to be interpolated, the blue dots represent the original sampling points, the black dotted and solid lines are the Voronoi diagrams generated based on the blue points, and the orange area is the new region $A\left(q\right)$ generated by point q according to the rule. A sampling point is a natural neighbor of q if its original region is overlapped by this orange area. The value of point q is the weighted average of the values of all neighboring points, and the interpolation weight $\omega_{i}$ is directly proportional to the overlapping area $A\left(p_{i}\right)$ borrowed by point q from the i-th neighbor. Therefore, the weight $\omega_{i}$ of the i-th neighbor $p_{i}$ is the following Equation (2):

(2) $$\omega_{i}=\frac{A\left(p_{i}\right)}{A\left(q\right)}$$

The final interpolation result $f\left(q\right)$ is the following Equation (3):

(3) $$f\left(q\right)=\sum_{i=1}^{n}\omega_{i}\left(q\right)f\left(p_{i}\right)$$

This method is $C^{1}$ continuous (smooth) everywhere except at data points and, like linear interpolation, strictly bounds results within sample values. It avoids the global equation solving required by splines and is governed by local neighborhoods, the value of the interpolation point is determined by who originally owned the space it occupies. The more area it occupies of that original point, the greater its weight will be.

Given the physical constraints of wellbore multiphase flow, natural neighbor interpolation is the core algorithm chosen for this study. It combines the smoothness of cubic interpolation with the numerical stability of linear interpolation.

## 5. Results and Discussion

**Figure 11:**
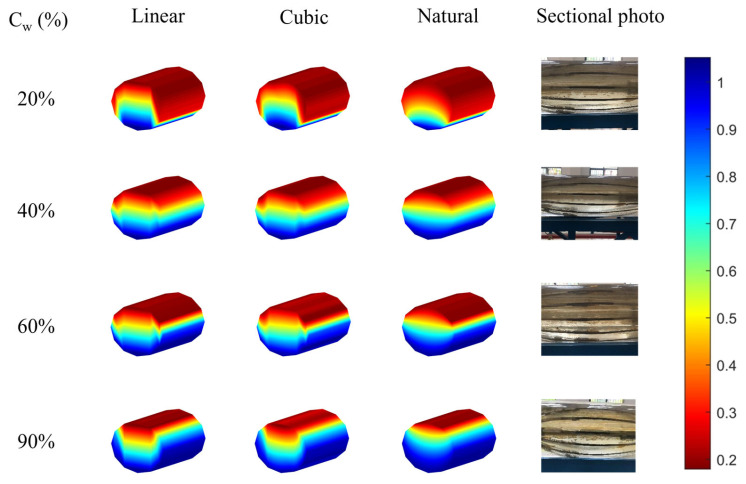
The imaging results of different interpolations under different water cuts of 100 m^3^/d were compared with the actual photos.

As shown in Figure 11, when the flow rate is 100 m^3^/d, due to the low flow velocity, the oil–water two-phase mixture naturally separates under the influence of gravity, forming a relatively smooth and stable stratified flow (ST), with only a small amount of oil droplets being observed in the water phase. 

By comparing the imaging effects of the three interpolation algorithms, it was found that the results of linear interpolation and cubic spline interpolation have similar limitations. At water cuts of 20% and 90%, these two algorithms are overly sensitive to the sensor response at the phase interface, easily causing steep non-physical mutations, resulting in significant protrusions or depressions in the interface morphology; at water cuts of 40% and 60%, although the overall imaging is acceptable, oscillation phenomena in the interpolation data occur at the phase interface and the wellbore boundary.

**Figure 12:**
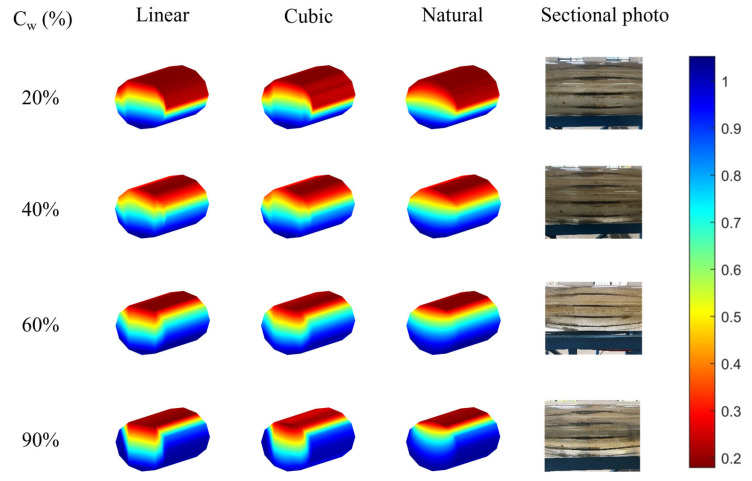
The imaging results of different interpolations under different water cuts of 300 m^3^/d were compared with the actual photos.

**Figure 13:**
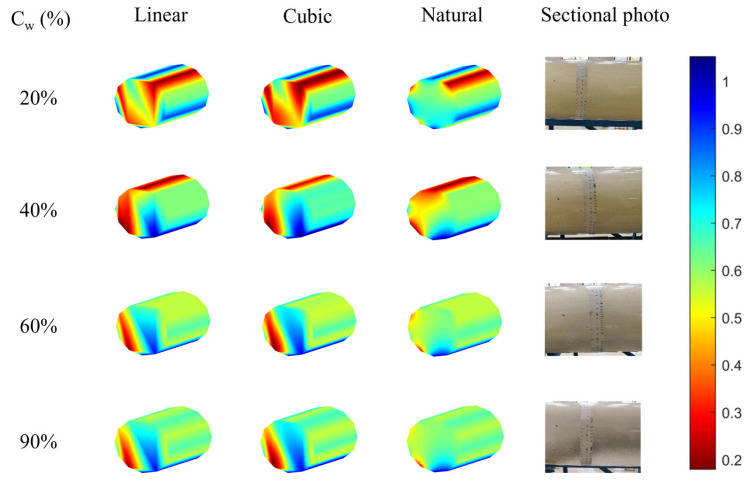
The imaging results of different interpolations under different water cuts of 600 m^3^/d were compared with the actual photos.

Three interpolation methods were applied to 90° deviation data. Table 2, Table 3 and Table 4 show sensor responses, and Figure 11, Figure 12 and Figure 13 display imaging results (blue = water; red = oil).

In contrast, the natural neighbor point interpolation shows excellent stability under all water cuts, and the imaging results are very close to the actual cross-section photographs taken in the laboratory. Thanks to its neighborhood weighted mechanism based on Voronoi diagram reconstruction, this algorithm effectively overcomes the problems of steep turns and boundary oscillations, generating a smoother and more realistic fluid distribution image that accurately reflects the multiphase flow structure in the wellbore.

As shown in Figure 12, as the flow rate increases to 300 m^3^/d, the fluid turbulence in the wellbore becomes more significant. From the laboratory profile photographs, it can be seen that at a 20% water cut, a large number of oil foams and bubbles accumulate at the oil–water interface, forming a stratified flow with mixing interface (ST & MI). When the water cut rises to 40% and 60%, the shearing action intensifies. Part of the oil phase breaks into bubbles and disperses into the water, while water droplets are also entrained into the oil phase, forming a bidirectional dispersed foam flow. When the water cut reaches 90%, the oil fraction is significantly reduced and sheared into discrete droplets by the high-speed water flow, forming a typical dispersed flow.

Based on the interpolation results, the interface mixing effect caused by high flow rates has changed the performance of the algorithm. Interestingly, at a 20% water cut, linear interpolation and cubic spline interpolation did not show the steep transitions seen at low flow rates. This is mainly because a “gas–liquid mixed transition zone” was formed at the interface. This transition zone smoothed the gradient of fluid properties spatially, allowing the sensor readings to transition more smoothly and avoiding the “over-reaction” of the algorithm. However, at a water cut of 90%, due to the scarcity of the oil phase and the lack of a thick mixed layer, the sensor once again captured sharp responses, causing linear and cubic interpolations to produce unrealistic distortions. In conclusion, although the mixing effect improved the performance of linear and cubic interpolations to a certain extent under specific conditions, natural neighborhood interpolation, due to its strong structural reconstruction ability, can maintain the best imaging quality under all conditions of 300 cubic meters per day.

As shown in Figure 13, when the flow rate surges to 600 m^3^/d, the fluid dynamic characteristics change drastically. At a 20% water cut, the high-speed oil phase dominates and squeezes the water phase toward the well wall, forming a typical water-in-oil emulsion flow (w/o). As the water cut increases to 40% and 60%, strong turbulence takes over. Most of the oil phase is dispersed by the water flow, and the wellbore exhibits a foam flow morphology with intense oil–water interlocking. When the water cut reaches 90%, the strong shear force generated by the high flow velocity completely breaks the sparse oil phase into fine droplets, and the flow pattern transforms entirely into dispersed flow.

The imaging results clearly indicate that under the high flow rate of 600 m^3^/d, the flow field structure is extremely complex. It has deviated from simple stratified flow and evolved into a mixed system containing emulsion flow, foam flow, and dispersed flow. When dealing with such a heterogeneous and highly turbulent flow field, the natural neighbor interpolation demonstrates clear advantages. Compared with linear and cubic interpolations, it can more realistically reconstruct the complex topological structure of interwoven oil bubbles and water phases in the wellbore. It accurately captures the local features of the mixing region; therefore, it has higher imaging fidelity.

## 6. Conclusions

Accurate measurement and visualization of water holdup are of paramount importance for revealing fluid distribution laws and calculating flow parameters in horizontal wells. Based on calibration data from the Capacitance Array Tool (CAT) and combined with classic horizontal well oil–water two-phase flow regime maps, this paper analyzes the mechanism of flow regime evolution under experimental conditions in depth. Addressing the limitations of existing research in 3D imaging of horizontal wells, this study innovatively introduces three interpolation algorithms to reconstruct the 3D oil–water two-phase flow within the wellbore and systematically evaluates the imaging quality and applicability of each algorithm.

From the correlation between the imaging results and the fluid flow mechanism, it can be seen that the natural neighbor point interpolation algorithm has significantly higher accuracy in the three-dimensional imaging of horizontal wells than the linear and cubic spline interpolation algorithms, but its computational cost is correspondingly higher—about four times that of the other two algorithms. Under low flow conditions, the flow field interface is clear and smooth, the phase stratification is stable (oil phase on top, water phase at the bottom), the spatial gradient of the cross-sectional phase distribution is gentle, and there are no local mutations. At this time, the linear interpolation or cubic spline interpolation algorithm is preferred—both are based on the geometric logic of Delaunay triangulation and can effectively fit the continuous and smooth phase distribution characteristics, have high computational efficiency, and are well adapted to the low complexity flow field. In medium and high flow conditions, the flow field interface will form a “gas–liquid mixed transition zone” containing dispersed oil bubbles and water droplets (or the interface even disappears). The cross-section presents a highly non-uniform mixed system, the spatial gradient is intense, and there are random local mutations. At this time, the linear and cubic spline interpolation are prone to generate data oscillations due to the discontinuity of the property gradient in the interface area, while the natural neighbor interpolation relies on the neighborhood weighting mechanism of Voronoi diagram, which can adapt to the topological changes in the mixed area, effectively suppress non-physical mutations, and is more suitable for capturing the interface mixing characteristics.

Comparison of combined imaging results: When the flow rate is 100 m^3^/d, under the conditions of 40% and 60% water cut, the imaging effects of linear and cubic spline interpolation are better; however, when the water cut is 20% and 90%, distortion occurs, and the natural neighboring point interpolation algorithm needs to be used instead; when the flow rate is 300 m^3^/d, only in the case of excessively high water cut will the linear and cubic spline interpolation show distortion; when the flow rate rises to 600 m^3^/d, the imaging of linear and cubic spline interpolations is completely distorted (manifested as a large area of a single phase state within the wellbore), while the imaging effect of the natural neighboring point interpolation is better. Compared with this, the imaging effect of natural neighboring point interpolation is superior, but it still cannot clearly distinguish the specific fluid flow state, being only able to clearly identify it as a mixed phase state.

In summary, in low flow rate conditions, three-dimensional wellbore imaging can be directly carried out; in medium and low flow rate conditions, when the water cut is too low or too high, a more accurate natural neighboring point interpolation algorithm needs to be used; when the water cut is moderate, linear or cubic spline interpolation algorithms with higher computational efficiency can be selected; and in high flow rate conditions (such as frothy flow, emulsified flow), three-dimensional wellbore imaging is currently not applicable.

Considering the differences between the laboratory environment and the actual underground conditions, the flow patterns inferred based on the current profile observations may have certain limitations. Future research should focus on the following directions: First, expand the range of experimental variables to further broaden the coverage of flow rate and water cut in order to enhance the universality of the research conclusions; second, optimize the algorithm running efficiency to break through the existing computational bottlenecks, and extend the imaging capability of the current local well section to continuous three-dimensional imaging of the entire wellbore, providing more complete support for experimental verification and result analysis; third, through the coupling of multiple data sources, constrain and calibrate the foamy flow and emulsified flow under high flow rate conditions, striving to restore the true flow state of the fluid to a certain extent and making up for the deficiencies of the existing imaging methods in the characterization of complex flow patterns.

The three-dimensional wellbore imaging method proposed in this paper differs fundamentally from the existing two-dimensional cross-sectional imaging in that it breaks through the limitation of single-point local observation. Two-dimensional imaging can only obtain a snapshot of the phase distribution in a single section and is unable to capture the axial evolution characteristics of the flow field, while this study’s three-dimensional imaging, through continuous sampling along the well axis and three-dimensional reconstruction, albeit only for the fluid flow in a 1-meter-long stable well section, has constructed a technical framework that can fully present the axial transformation of the flow pattern, the migration of the phase distribution, and the dynamic changes in complex flow structures, providing more comprehensive experimental support for analyzing the evolution mechanism of the axial flow field, accurately quantifying the critical conditions for flow pattern transformation, and calculating the flow rate. Subsequent research will be based on this framework, expanding to the three-dimensional imaging of longer well sections and combining artificial intelligence algorithms to achieve refined imaging characterization of fluid flow in high-flow conditions.

Wellbore water holdup imaging analysis is directly related to oilfield production efficiency and resource evaluation. Although this paper has achieved certain results under ideal experimental conditions, challenges remain in the face of complex actual downhole environments. Looking ahead, with the deep integration of IoT, big data, and artificial intelligence technologies, water holdup monitoring is bound to develop towards intelligence, real-time capability, and adaptivity. Meanwhile, the development of novel non-intrusive measurement techniques to reduce operational interference and costs, combined with refined 3D interpolation imaging technology, will provide strong technical support for the precise interpretation of production profiles and efficient oilfield development.

## Figures and Tables

**Table 1:** Oil–water two-phase flow experimental scheme.

Angle of Inclination: 60°, 85°, 90°, 93°					
Logging Speed: 10 m/min, 15 m/min, 20 m/min					
Flow (m^3^/d)	Water Cut (%)	Flow (m^3^/d)	Water Cut (%)	Flow (m^3^/d)	Water Cut (%)
100	20	300	20	600	20
	40		40		40
	60		60		60
	90		90		90

**Table 2:** The 100 m^3^/d CAT sensor response value with different water cuts.

	C_w_ (%)	20%	40%	60%	90%
Number of CAT Sensors					
1		0.18	0.19	0.26	0.27
2		0.22	0.2	0.22	0.22
3		0.365	0.33	0.2	0.55
4		0.415	0.67	0.34	0.83
5		0.65	0.935	0.98	0.99
6		1	0.98	0.99	0.99
7		1.04	1	0.99	1
8		1	0.99	1	0.99
9		0.65	0.941	0.99	0.99
10		0.415	0.65	0.2	1
11		0.37	0.32	0.25	0.21
12		0.2	0.22	0.25	0.28

**Table 3:** The 300 m^3^/d CAT sensor response value with different water cuts.

	C_w_ (%)	20%	40%	60%	90%
Number of CAT Sensors					
1		0.21	0.2	0.21	0.21
2		0.2	0.19	0.22	0.18
3		0.22	0.36	0.2	0.82
4		0.25	0.754	0.34	0.98
5		0.83	0.91	0.98	1
6		1	0.98	0.99	0.99
7		0.99	0.99	0.99	1
8		1	0.99	1	1
9		0.847	0.97	0.99	1.01
10		0.51	0.77	0.2	0.97
11		0.22	0.398	0.25	0.972
12		0.23	0.34	0.25	0.21

**Table 4:** The 600 m^3^/d CAT sensor response value with different water cuts.

	C_w_ (%)	20%	40%	60%	90%
Number of CAT Sensors					
1		0.97	0.96	0.98	0.98
2		0.59	0.98	0.99	0.99
3		0.79	0.98	0.98	0.98
4		0.82	0.98	0.99	0.99
5		0.95	0.985	0.98	0.98
6		0.76	1	1.02	1.02
7		1.03	1	1.03	1.03
8		0.66	0.96	0.94	0.94
9		0.98	0.96	0.99	0.99
10		0.9	0.99	0.98	0.98
11		0.62	0.97	0.96	0.97
12		0.95	0.97	0.99	0.99

## Data Availability

The relevant data has been disclosed in the main text. More data associated with this research are available and can be obtained by contacting the author.
